# Clear Cell and Histiocytic/Dendritic Cell Sarcomas: Clinical Outcomes, Molecular Features, and Diagnostic Pitfalls

**DOI:** 10.3390/cancers18040641

**Published:** 2026-02-16

**Authors:** Gabriel Tinoco, Marium Husain, David Liebner, James L. Chen, Swati Satturwar, Hans Iwenofu, Valerie Grignol, Joal Beane, Scott Lenobel, David Konieczkowski, Carl Quinion, Joel Mayerson

**Affiliations:** 1 Division of Medical Oncology, Department of Internal Medicine, The Ohio State University Comprehensive Cancer Center, Columbus, OH 43210, USA; marium.husain@osumc.edu (M.H.); david.liebner@osumc.edu (D.L.); james.chen@osumc.edu (J.L.C.); 2 Department of Pathology, The Ohio State University Comprehensive Cancer Center, Columbus, OH 43210, USA; swati.satturwar@osumc.edu (S.S.); hans.iwenofu@osumc.edu (H.I.); 3 Division of Surgical Oncology, Department of Surgery, The Ohio State University Comprehensive Cancer Center, Columbus, OH 43210, USA; valerie.grignol@osumc.edu (V.G.); 4 Department of Radiology, The Ohio State University Comprehensive Cancer Center, Columbus, OH 43210, USA; 5 Department of Radiation Oncology, The Ohio State University Comprehensive Cancer Center, Columbus, OH 43210, USA; 6 Department of Orthopedic Surgery, The Ohio State University Comprehensive Cancer Center, Columbus, OH 43210, USA

**Keywords:** histiocytic sarcoma, dendritic cell sarcoma, clear cell sarcoma, ultra-rare sarcomas, clinical outcomes, molecular profiling

## Abstract

**Background:** Dendritic and histiocytic cell sarcoma (DHCS) and clear cell sarcoma (CCS) are ultra-rare soft-tissue sarcomas characterized by diagnostic ambiguity, limited treatment guidelines, and poor outcomes. Their rarity has restricted the development of evidence-based management strategies, leaving clinical decisions reliant on small case series and institutional experience. DHCS typically presents without a unifying molecular driver and is often misclassified without comprehensive immunophenotyping. CCS is defined by EWSR1-ATF1/CREB1 fusions but exhibits low responsiveness to conventional chemotherapy. There remains a clear need to clarify natural history, therapeutic responses, and molecular characteristics in both. **Methods:** We conducted a retrospective cohort study of adult patients with histologically confirmed DHCS or CCS seen at The Ohio State University Comprehensive Cancer Center between 2010 and 2022. Demographics, treatment modalities, clinical outcomes, and molecular profiles were extracted and analyzed descriptively. Time to progression (TTP) and progression rates by treatment modality were recorded. A structured literature review was conducted to provide context for the findings. **Results:** Outcomes are descriptive and cohort-specific, reflecting institutional experience rather than generalizable estimates. A total of 10 patients with DHCS and 5 with CCS were evaluable. Most DHCS patients presented with metastatic disease. Among DHCS patients who received systemic therapies, 5 of 8 (62.5%) experienced progression during or shortly after treatment. Among CCS patients who received systemic therapies, 3 of 4 (75%) progressed during or shortly after treatment. Overall mortality occurred in 4 of 10 DHCS patients (40%) and 3 of 5 CCS patients (60%). TP53 mutations were identified in 4 of 7 next-generation sequencing (NGS)-tested DHCS cases, and PD-L1 positivity was detected in 5 of 7 tested DHCS cases and 1 of 5 tested CCS cases. **Conclusions:** Despite multimodal treatment, this referral-based cohort of patients with ultra-rare DHCS and CCS showed high rates of progression and mortality. Our findings underscore the urgent need for multi-institutional collaboration and biomarker-driven clinical trials to guide management of these ultra-rare sarcoma subtypes.

## Simple Summary

Dendritic and histiocytic cell sarcoma (DHCS) and clear cell sarcoma (CCS) are ultra-rare cancers that are difficult to diagnose and lack clear treatment guidelines, often leading to delayed recognition and inconsistent care. In this retrospective study of adults treated at a tertiary sarcoma center between 2010 and 2022, most patients presented with advanced disease and received multimodal management with surgery, radiation, and systemic therapies. This experience represents one of the larger single-institution DHCS series and a contemporaneous CCS cohort, with more granular line-level treatment and outcome data than many prior reports. However, over half of patients progressed under systemic therapy, and mortality remained high (40% in DHCS and 60% in CCS), highlighting poor outcomes observed with currently used approaches in this referral-based cohort. Molecular profiling of tumor protein p53 (TP53), programmed death-ligand 1 (PD-L1), and EWS RNA binding protein 1 (EWSR1) fusions provides biomarker-relevant data supporting the need for expert pathology review; systematic testing; and enrollment in collaborative, biomarker-driven clinical trials.

## 1. Introduction

Soft-tissue sarcomas (STS) comprise a biologically diverse family of mesenchymal tumors spanning nearly 100 histologic and molecular entities [1]. They are uncommon in adults, accounting for roughly 1% of all cancers in the United States, with an estimated 13,500 new diagnoses in 2020 [2]. Care is typically multimodal, integrating systemic therapy with surgery and, when appropriate, radiation therapy [3,4]. Despite therapeutic progress, outcomes for metastatic STS remain poor: about half of patients die within 18 months of presentation [1,2,5].

Ultra-rare sarcomas are subtypes with an annual incidence of about 1 per 1 million people or less. This incidence-based threshold, proposed by Silvia Stacchiotti and the Connective Tissue Oncology Society (CTOS) consensus group, was set to flag entities so uncommon that conventional, histology-specific prospective trials are rarely feasible. Using this criterion, the group cataloged 56 ultra-rare types in soft tissue and 21 ultra-rare types in bone, together representing roughly one-fifth of all sarcomas [6]. The optimal treatment of ultra-rare sarcomas remains unclear.

Dendritic and histiocytic cell sarcoma (DHCS) and clear cell sarcoma (CCS) are ultra-rare sarcomas with diagnostic complexity, uncertain therapeutic standards, and scant prospective evidence. Current management is not standardized: DHCS care is individualized (surgery when feasible; heterogeneous systemic therapy in advanced disease) [7], and CCS relies on surgery for localized tumors, with limited activity of conventional chemotherapy in the metastatic setting [8], leaving a clear treatment gap for both entities [9]. Misclassification remains a recognized challenge.

We present a single-center experience that, to our knowledge, represents a meaningful institutional cohort for each entity, and synthesize the current literature to highlight diagnostic pitfalls; outcomes under contemporary care; and opportunities for rational, biomarker-anchored therapy within master-protocol and rare-disease research networks. DHCS and CCS were examined together in this study because both represent ultra-rare, aggressive sarcoma subtypes with overlapping clinical challenges, including diagnostic complexity, limited prospective data, and the absence of standardized treatment paradigms.

### 1.1. DHCS

DHCSs are neoplasms affecting monocyte–macrophage and dendritic cell lineages. They present across nodal and extranodal sites and exhibit a histiocytic morphology with supportive immunophenotypes (commonly, CD163, CD68, lysozyme, and PU.1); negative lineage markers are essential to exclude mimics [7]. Diagnosis is challenging because there is no single pathognomonic molecular signature, morphologic appearances vary, and overdiagnosis has been reported without a broad immunopanel and clinicopathologic correlation [10]. Population data suggest histiocytic sarcoma has an age-adjusted incidence of ~0.17 per 1 million/year, with a median overall survival (OS) of approximately 6 months and a 5-year disease-specific survival of about 40%, underscoring its aggressive course [11]. DHCS classification and biology continue to evolve as more cases are studied, and recent consensus work has emphasized the importance of clear diagnostic standards for these ultra-rare sarcomas [12].

DHCS most commonly arises in lymph nodes, particularly in the head and neck region (60% in our series), but may also involve extranodal sites such as bone, skin, and soft tissue and often presents with advanced or disseminated disease. Its diagnosis is challenging due to histologic overlap with other histiocytic and dendritic cell neoplasms, high-grade lymphomas, and poorly differentiated sarcomas. Differential diagnosis includes follicular dendritic cell sarcoma, histiocytic sarcoma, lymphoma, and carcinoma [13].

Molecular studies show that histiocytic and dendritic cell neoplasms vary widely in their biology. Recurrent alterations in the RAS-RAF-MAPK pathway, including KRAS, NRAS, MAP2K1, and BRAF mutations, have been described in subsets of histiocytic sarcoma. These findings help distinguish histiocytic sarcomas, which often harbor MAPK pathway alterations, from follicular dendritic cell sarcoma, which more frequently shows homologous recombination deficiency. These biologic differences support ongoing efforts to tailor treatment based on molecular features when present [7,14,15].

For localized DHCS, we pursue complete surgical excision; radiotherapy may be considered for close or positive microscopic margins. In advanced disease, there is no consensus regimen; practice patterns include anthracycline-based sarcoma therapy or lymphoma-style chemotherapy; in selected molecular contexts, MAPK-directed therapy has been used, though evidence remains limited to small series and case reports [7,9,16,17].

Clinical experience remains limited, and published outcomes highlight both the rarity and heterogeneity of these tumors. Large, pooled analyses and multi-institutional series describe varied presentations involving nodal and extranodal sites, frequent misclassification, and inconsistent responses to systemic therapy. Across these reports, surgery remains the most consistent component of therapy for localized disease, while systemic therapy and radiation are used selectively in advanced or unresectable cases [13,18,19].

The gap is both diagnostic and therapeutic: no single genetic hallmark defines all cases, histologic mimics are common, prospective trials are lacking, and outcomes remain variable and often suboptimal under repurposed treatment regimens [7].

Although all cases in this series were reviewed by subspecialty pathologists at our institution using integrated histologic, immunophenotypic, and molecular criteria, the absence of a defining molecular hallmark for DHCS and overlapping features with other malignancies continue to pose substantial diagnostic challenges, particularly outside specialized centers.

### 1.2. CCS

CCS is an ultra-rare, translocation-driven STS that typically arises in young adults, often as a slow-growing mass in the distal extremities near tendons/aponeuroses. It lacks sex predilection, occurs more frequently in White patients, and is defined by EWSR1-ATF1 or EWSR1-CREB1 fusions that are pathognomonic and critical for distinguishing it from melanoma. Surveillance, Epidemiology, and End Results (SEER) program-based analyses estimate an incidence of about 0.012–0.027 per 100,000/year (≈0.12–0.27 per million). Reported survival varies by cohort and stage; population data show 1-, 3-, and 5-year OS to be approximately 78%, 62%, and 57%, respectively, while other series cite ~50% 5-year and ~38% 10-year OS, reflecting the poor long-term outlook when disease is advanced [20].

CCS most frequently arises as a deep soft-tissue tumor of the distal extremities in adolescents and young adults, characterized by EWSR1–ATF1 or EWSR1–CREB1 fusions. Importantly, no renal CCS cases were identified in the present cohort, as clear cell sarcoma of the kidney (CCSK) represents a separate pediatric renal malignancy that is molecularly distinct from soft-tissue CCS (EWSR1-negative). The differential diagnosis includes malignant melanoma, synovial sarcoma, perineurioma, and other translocation-associated neoplasms [21].

CCS carries a transcriptional and immunophenotypic profile that overlaps with melanoma, including the expression of S100, SOX10, and PRAME. This overlap can complicate diagnostic distinction from melanoma, particularly in small biopsies. Reviews of CCS pathology and management note that accurate diagnosis requires the integration of morphology, immunohistochemistry (IHC), and confirmation of EWSR1 rearrangements [22].

Localized CCS is managed with wide surgical excision in experienced sarcoma centers; radiotherapy is employed selectively for local control. In advanced disease, conventional chemotherapy has low response rates; limited activity of VEGFR and MET inhibitors has been reported in advanced CCS, including modest responses to sunitinib and pazopanib in retrospective series, but there is no established systemic standard [8,9].

Conventional chemotherapy has low response rates, and the role of systemic therapy remains inadequately defined due to the very limited availability of prospective data. Emerging strategies include the use of VEGFR/MET tyrosine kinase inhibitors and early efforts to target fusion-driven transcriptional programs or epigenetic co-dependencies, as well as the use of checkpoint inhibitors [8,23,24,25].

Accordingly, we aim to characterize the clinicopathologic features, management patterns, and outcomes of DHCS and CCS and to integrate these findings with a structured literature review to identify diagnostic pitfalls and therapeutic signals to guide future trial design.

## 2. Methods

### 2.1. Study Design and Oversight

We conducted a single-center, retrospective cohort study at The Ohio State University Comprehensive Cancer Center—James (OSUCCC-James), evaluating patients with DHCS and CCS. The study covered 1 January 2010 through 1 April 2022. The protocol was approved by the OSU Institutional Review Board (IRB protocol #: 2022C0063–2022C0062).

### 2.2. Setting

OSUCCC-James is a tertiary academic referral center with a dedicated sarcoma program, integrated pathology and molecular diagnostics, and enterprise electronic health records (EHRs; Epic). Radiology images and reports are archived in an institutional picture archiving and communication system (PACS).

### 2.3. Cohort Identification and Eligibility

We queried institutional tumor registries, pathology databases, and EHRs to identify adult patients (≥18 years) with a diagnosis code or pathology report consistent with DHCS or CCS during the study window. Final inclusion required histologic confirmation of DHCS or CCS by subspecialty hematopathology and sarcoma pathologists, incorporating morphology; immunohistochemistry; and, when available, molecular testing, consistent with contemporary WHO criteria. Cases identified as misclassified during this institutional pathology review were excluded from the analytic cohort. Inclusion criteria included histologically confirmed DHCS or CCS; first encounter at OSUCCC-James within the study period; and availability of core clinical data including demographics, diagnostic pathology, disease extent at presentation, treatment details, and follow-up outcomes. We excluded pediatric patients (<18 years), patients with a non-OSU index diagnosis and no meaningful clinical data or follow-up at OSU, misclassification on pathology re-review, and duplicate records. DHCS diagnosis required characteristic morphology supported by immunohistochemistry and exclusion of mimics (no unifying molecular driver required); CCS required morphology/immunophenotype with EWSR1 rearrangement confirmation when tissue available (4/5 cases, 80%). Specifically, DHCS diagnosis relied on morphology, immunohistochemistry, and exclusion of mimics (no unifying molecular driver required); CCS diagnosis included confirmation of EWSR1 rearrangement using fluorescence in situ hybridization (FISH) and/or next-generation sequencing (NGS). when tissue available.

### 2.4. Data Sources and Variables

We abstracted structured and unstructured data from the EHR into a Research Electronic Data Capture (REDCap; Vanderbilt University, Nashville, TN, USA) database (or secured spreadsheet) with pre-specified fields and data dictionaries. Collected baseline variables included demographics (age, sex, and race/ethnicity), tumor factors (primary site, size (largest dimension on baseline imaging or pathology)), disease extent, date of diagnosis, and pathology (histologic subtype, IHC markers, fusion results (CCS), and other ancillary tests). Collected treatment variables included modalities—surgery, radiotherapy, and systemic therapy (regimen names, doses, cycles, start/stop dates, and reasons for discontinuation); for systemic therapy lines, we recorded sequence order and intent (curative vs. palliative). The following clinical outcomes were recorded: time to progression (TTP) for each systemic therapy line, i.e., start date of the line to radiographic or unequivocal clinical progression, death, or last disease assessment (whichever came first); progression status per line and per modality (yes/no); OS, i.e., diagnosis to death from any cause (censored at last known alive); and vital status at last follow-up.

Because systemic regimens, treatment intent, and sequencing varied across patients, progression proportions are reported descriptively per line of therapy and reflect outcomes following treatment exposure rather than treatment efficacy. Comparisons across modalities or regimens were not performed and are not supported by the study design.

We also collected molecular and immune markers as reported by clinical laboratories. Molecular profiling reflects real-world testing performed as part of routine clinical care rather than protocol-driven molecular characterization and consisted of targeted, DNA-based next-generation sequencing panels obtained through multiple platforms, including commercially available assays (predominantly Tempus xT/xE panels; Tempus Labs, Chicago, IL, USA, and FoundationOne panels; Foundation Medicine, Cambridge, MA, USA), one Caris assay (Caris Life Sciences, Irving, TX, USA), and institutional in-house targeted sequencing panels. Institutional testing utilized polymerase chain reaction (PCR)-based AmpliSeq libraries (Thermo Fisher Scientific, Waltham, MA, USA) sequenced on Ion S5 or Ion Torrent Genexus platforms (Thermo Fisher Scientific, Waltham, MA, USA), with variant analysis performed using standard clinical pipelines and final interpretation rendered by subspecialty molecular pathologists integrating clinicopathologic context. Panel content varied by platform and time period. Tumor mutational burden (TMB) was recorded as reported (mutations per megabase, mut/Mb), recognizing variability across panels, and programmed death-ligand 1 (PD-L1) expression was assessed by immunohistochemistry when available (assay and scoring system recorded if specified). No comprehensive genomic, whole-exome, whole-genome, or transcriptomic (expression-based) analyses were conducted.

Imaging (CT/MRI) and reports were reviewed. Where available, the response was abstracted by the treating physician using the Response Evaluation Criteria in Solid Tumors (RECIST) v1.1 criteria. If RECIST calls were not documented, we used clinician-documented progression language corroborated by imaging dates. Imaging intervals varied by clinical practice; typical restaging occurred every 8–12 weeks for advanced disease.

Two abstractors performed data entry with 10–20% dual review to assess agreement; discrepancies were resolved by consensus with investigator oversight. Missingness was summarized per variable; no imputation was performed. Dates were checked for internal consistency (diagnosis → treatment → follow-up), and outliers were verified against source notes.

### 2.5. Outcomes and Analysis Plan

Given the small sample size and heterogeneity, the analysis was primarily descriptive.

- Descriptive statistics: counts/percentages and medians with interquartile ranges (IQR) for continuous data.
- Systemic therapy/radiotherapy surgery progression proportions: numerator = number progressed; denominator = number treated with that modality.
- Time-to-event analyses: Kaplan–Meier estimation for OS and, when feasible, for progression-free survival (PFS)/TTP at the patient level; medians with 95% confidence intervals (CIs). Where there were too few events, we reported event counts and follow-up times.
- For clarity, progression proportions by treatment modality are reported at the patient level (number of patients experiencing progression among those exposed), whereas TTP is assessed per line of therapy.
- Exploratory associations: Where cell sizes permitted, exact tests (Fisher’s) for categorical associations and non-parametric tests for continuous measures (e.g., Wilcoxon). No formal multiple-comparison adjustment is given, considering the hypothesis-generating scope.

Analyses were performed in R (version 4.5.0; R Foundation for Statistical Computing, Vienna, Austria). A 2-sided alpha of 0.05 was used for descriptive *p*-values, with appropriate caution about inferential limits.

### 2.6. Literature Review

We performed a structured (non-meta-analytic) literature review to contextualize diagnostics, management patterns, and outcomes for DHCS and CCS using PubMed/MEDLINE and ClinicalTrials.gov from 1 January 2010 to 30 August 2025, limiting the results to English-language and human studies. The following keywords were used in our search:

dendritic cell sarcoma OR histiocytic sarcoma OR interdigitating dendritic cell sarcoma OR Langerhans cell sarcoma AND clinical OR outcomes OR treatment OR chemotherapy OR surgery OR radiotherapy OR targeted OR immunotherapy

clear cell sarcoma AND EWSR1 OR ATF1 OR CREB1 OR fusion OR translocation OR outcomes OR systemic therapy

We included original clinical data (prospective/retrospective cohorts, case series ≥ 5 patients); systematic reviews for background; and case reports, which were used sparingly to illustrate feasibility or signals in ultra-rare contexts. Two reviewers screened titles and abstracts, then full texts. Any disagreements were resolved by consensus. We extracted study design, population, diagnostic criteria, treatments, and outcomes (response, PFS, and OS). Study quality and risk of bias were judged qualitatively (selection, misclassification, and confounding).

## 3. Results

Given the very small cohort size, all outcome proportions are reported descriptively and should not be interpreted as representative estimates of progression or survival for these sarcoma subtypes.

The detected genomic alterations were heterogeneous and identified in the context of limited, real-world targeted sequencing; as such, their functional significance and role as disease-defining driver events cannot be determined from this dataset.

### 3.1. DHCS

A total of 18 patients with DHCS were identified. 8 patients were excluded from analysis due to loss to follow-up or receipt of care outside our institution, leaving 10 evaluable patients. Of these, 1 (10%) was Black, 9 (90%) were White, 7 (70%) were male, and 3 (30%) were female. The median age at diagnosis was 54 years (range, 24–67). The most common primary tumor site was the head and neck region (60%), and the average tumor size was 4.8 cm.

At initial presentation, 4 patients (40%) had localized disease, and 6 (60%) had metastatic disease. Systemic therapies were administered to 8 patients, 3 underwent radiation therapy (with or without sequential chemotherapy), and 3 had surgical resection (R0/R1 margins not uniformly documented).

Patients receiving systemic therapies underwent 2 to 6 cycles using various regimens—most commonly, cytotoxic agents.

A total of 10 patients received a total of 14 lines of systemic therapy, reflecting that several individuals progressed through or were transitioned between more than one regimen over the course of their disease. Accordingly, the number of treatment lines exceeds the number of unique patients, and progression proportions are reported per line of therapy rather than per patient to more accurately capture treatment exposure and observed outcomes.

These proportions represent patterns of response and failure observed during treatment exposure and should not be interpreted as comparative measures of efficacy between modalities or regimens.

TTP was assessed for each line of therapy. The shortest TTP was 16 days in a patient treated with single-agent sirolimus. The longest TTP was 354 days (51 weeks) in a patient who received cyclophosphamide, doxorubicin, and vincristine. Due to the small sample size, progression rates were reported instead of median TTP. Among patients treated with systemic therapies, 5 of 8 (62.5%) experienced disease progression, compared to 1 of 3 (33.3%) among those treated undergoing radiation or surgery.

4 of the 10 evaluable patients (40%) died from the disease during the study period. Somatic NGS was performed in 7 of 18 patients. TP53 mutations were identified in 4 cases. PD-L1 expression was assessed by immunohistochemistry (IHC) in these 7 patients, and 5 showed positive PD-L1 staining. The median tumor mutational burden was 4.75 mutations per megabase (range, 3–127). The upper-range TMB value may reflect technical variability or biological outlier status, given panel heterogeneity; sarcoma TMB values rarely exceed 15–20 mut/Mb in a validated dataset. Given the non-uniformity of testing and assay variability, these molecular and immune findings are reported descriptively and should be interpreted as exploratory rather than predictive.

### 3.2. CCS

A total of 5 patients with CCS were identified. 3 patients (60%) were White, and 2 (40%) were Black. The mean age at diagnosis was 46 years, with a median of 43 years (range, 29–66). 3 patients (60%) were female, and 2 (40%) were male. The mean tumor size at presentation was 5.62 cm. Of 5 diagnosed CCS cases, 2 patients (40%) had stage I disease, 1 (20%) had stage III, and 2 (40%) had stage IV disease. Treatment with curative intent was administered to 3 patients (60%). The primary tumor site was the abdomen, lower back, or a lower extremity in 4 of 5 patients (80%).

4 patients received systemic therapy, three underwent surgery with curative intent (R0/R1 microscopic margins not uniformly documented), and 2 received radiation therapy for local control. Systemic treatment consisted of heterogeneous systemic therapies, including cytotoxic chemotherapy and targeted agents, with patients receiving between 3 and more than 5 cycles per line of therapy. Among patients receiving systemic therapy, 3 of 4 (75%) progressed during or shortly after treatment. The shortest TTP was 30 days (4.3 weeks) in a patient treated with sunitinib. The longest TTP was 339 days (48 weeks) in a patient treated with pazopanib. Among early-stage patients who underwent surgery, 1 of 3 (33.3%) progressed. Neither of the 2 patients who received radiation therapy experienced progression. Given the heterogeneity of regimens and the small number of treated patients, this proportion reflects treatment exposure within this cohort and cannot be used to infer relative treatment efficacy.

3 patients (60%) died during the study period, including 2 with metastatic disease at diagnosis and 1 with localized disease. Targeted DNA-based next-generation sequencing using clinically ordered panels was performed in CCS cases when available, using the same general class of real-world clinical platforms as for DHCS. No recurrent pathogenic variants were identified across CCS cases, and observed alterations were heterogeneous. Tumor mutational burden values were variable and should be interpreted cautiously, given the assay heterogeneity and small sample size. One patient demonstrated positive PD-L1 expression by IHC. The median tumor mutational burden was 11.86 mutations per megabase (range, 2.06–28.16). As in DHCS, these results reflect heterogeneous clinical testing and are intended as descriptive observations rather than indicators of therapeutic sensitivity or biomarker-defined subgroups.

## 4. Conclusions

Published series describe a similar pattern of diagnostic difficulty and clinical variability across DHCS and CCS. Reports over the past decade highlight that these tumors often present at advanced stages, respond unpredictably to systemic therapy, and require pathology review by experienced centers. Treatment approaches differ widely across institutions, reflecting limited prospective evidence and reliance on retrospective cohorts [18,19].

In our retrospective series of DHCS and CCS, we found high rates of progression and substantial mortality despite the use of multimodal therapy, mirroring the aggressive clinical behavior previously reported in limited cohorts. For example, in an international retrospective series of 55 patients with CCS treated with systemic therapy, the median OS was approximately 15 months. Among those treated with sunitinib, the best objective response was 30% (*n* = 3 of 10), with a median PFS of 4 months [8]. Additional data confirm the limited efficacy of conventional systemic therapies in advanced CCS [8]. In DHCS, conventional cytotoxic chemotherapy has similarly demonstrated poor disease control: in prognostic analyses of histiocytic and dendritic cell neoplasms, OS remains modest, and relapse after systemic therapy is common [26]. These findings underscore the unmet need for targeted, immunotherapeutic, or biomarker-driven strategies in these ultra-rare sarcoma subtypes (see Table 1).

### 4.1. Molecular Insights and Therapeutic Implications

Interpretation of molecular findings in this series is inherently limited by reliance on heterogeneous, real-world targeted sequencing performed for clinical purposes rather than standardized comprehensive genomic or transcriptomic profiling. The molecular analyses in DHCS revealed TP53 mutations in four of seven tested DHCS cases and positive PD-L1 expression in several. Across both DHCS and CCS, the detected genomic alterations should be interpreted cautiously, as their identification through heterogeneous, real-world targeted sequencing does not allow for determination of whether they represent disease-defining driver events, secondary changes, or markers of genomic instability. Likewise, PD-L1 expression and TMB values in this series should be considered hypothesis-generating only, given non-uniform testing, assay heterogeneity, and the absence of outcome correlations. In other histiocytic neoplasms, PD-L1 positivity has correlated with responses to checkpoint inhibitors; for example, a patient with 100% PD-L1 expression DHCS achieved a sustained response to pembrolizumab [27]. The relevance of TMB remains uncertain: our medians (~4.75 in DHCS and ~11.9 in CCS) are low compared with highly immunogenic tumors; TMB global thresholds may not apply to ultra-rare sarcomas.

In CCS, EWSR1-ATF1/CREB1 fusion has been the molecular hallmark, but targetable downstream dependencies remain elusive. According to recent reviews, MET overexpression downstream of EWSR1 fusion has been investigated as a therapeutic target, although clinical responses to MET inhibitors have been limited [22,28]. Efforts to broadly harness immunotherapy in sarcoma are ongoing. A systematic review of immune checkpoint inhibitors (ICIs) in STS showed a pooled objective response rate of 14% and a disease control rate of 55% [29]. However, responses are highly histology-dependent, and the heterogeneity in immune profiles across sarcoma subtypes complicates generalization. The IMMUNOSARC II master trial [25] has already reported results in its CCS cohort using sunitinib plus nivolumab, which may provide early signal data relevant to both CCS and, potentially, DHCS.

Emerging case-level and real-world reports suggest that selected patients with histiocytic neoplasms may benefit from targeted or immune-directed therapies when supported by molecular or immunologic features. Responses have been reported with MEK inhibitors, BRAF-directed therapies, mTOR inhibition, and immune checkpoint blockade in patients with relevant pathway alterations or high PD-L1 expression. These experiences remain anecdotal and inconsistent but reinforce the value of molecular assessment in guiding treatment decisions [28,30,31,32,33,34,35]. Importantly, these findings derive from heterogeneous patient populations and are not directly generalizable to DHCS or CCS.

### 4.2. Future Directions

Our findings should be interpreted in light of several limitations, including the very small sample size, retrospective single-center design, and heterogeneous treatment approaches, which, together, preclude robust comparative or prognostic inference.

Treatment regimens, intent, and sequencing varied across patients, precluding meaningful comparison of outcomes across modalities or therapies. In addition, a detailed assessment of surgical margins and standardized local control outcomes was not feasible due to incomplete operative and pathology documentation and heterogeneity in radiation therapy records from referring institutions. As a result, interpretation of surgical and radiation outcomes is limited and reflects real-world referral practice in the management of ultra-rare sarcomas.

As a tertiary sarcoma referral center, our institution is preferentially referred patients with advanced, recurrent, or treatment-refractory DHCS and CCS, which likely contributes to the high progression and mortality observed. Because both entities meet consensus criteria for ultra-rare sarcomas and are often under-recognized or misclassified at presentation, some patients also experienced delays in definitive diagnosis and subspecialty management. Taken together, these factors indicate that the outcome proportions reported here reflect a referral-enriched, diagnostically complex cohort and should be viewed as institutional experience rather than population-level benchmarks for DHCS and CCS.

Our review of the literature was selective and narrative in scope. It prioritized peer-reviewed sources and clinical trial registries but was not conducted as a systematic review and was limited to English-language publications. As a result, relevant reports in non-indexed sources, non-English journals, or recent preprints may not have been captured. This limitation reflects the broader evidence landscape for ultra-rare sarcomas, where the literature is dominated by case reports and small case series and higher-quality evidence, such as randomized controlled trials, is largely unavailable.

Despite these constraints, this series provides detailed clinical and molecular data that may inform future prospective studies. Recurrent MAPK pathway alterations reported in histiocytic and dendritic cell neoplasms, emerging evidence of immune checkpoint pathway activation, and possible DNA damage response abnormalities suggest that MEK, ERK, or upstream kinase inhibitors; immune checkpoint blockade; and agents targeting DNA repair pathways may warrant evaluation in biomarker-selected subsets of DHCS and CCS. In CCS, transcriptional programs driven by EWSR1 fusions remain biologically compelling but incompletely understood therapeutic targets and may require indirect strategies focused on downstream signaling or epigenetic co-dependencies. For both DHCS and CCS, there is a paucity of robust preclinical models, and the development of genetically and immunologically faithful systems, including patient-derived xenografts and organoid platforms, will be essential to enable functional testing of candidate pathways. Broader enrollment in rare-sarcoma registries and collaborative networks will be essential to refine incidence estimates and validate candidate biomarkers.

The identification of potential therapeutic vulnerabilities (e.g., immune checkpoint pathways and DNA damage response) suggests the need to embed patients in biomarker-driven trials or umbrella protocols. Collaborative rare-sarcoma consortia (e.g., the World Sarcoma Network) are essential to enable accrual and translational correlative studies. In CCS specifically, prospective molecularly informed trials are long overdue, and integration of immune biomarkers, TMB, and tumor microenvironment analyses will be critical.

This study adds to the limited evidence base on DHCS and CCS by providing contemporaneous clinical and molecular data. The high rates of progression, modest survival outcomes, and limited benefit from standard therapies underscore the urgent need for novel approaches. While P53 alterations and PD-L1 expression patterns provide exploratory leads, their predictive and therapeutic significance requires prospective validation. However, these findings should be viewed as hypothesis-generating and descriptive of clinical experience with ultra-rare, diagnostically challenging sarcomas at a tertiary referral center rather than as definitive outcome data or population-level benchmarks for DHCS and CCS. Multidisciplinary collaboration, centralized pathology, and rare-disease trial networks will be pivotal. Ultimately, success will depend on the integration of molecular insights, immune profiling, and innovative trial designs to move beyond empiricism in these challenging sarcoma subsets.

## Figures and Tables

**Table 1:** Representative clinical series in clear cell and histiocytic/dendritic cell sarcomas.

Study/Data Source	Sample Size	Strengths	Limitations
OSU DHCS Cohort	10 evaluable patients (from 18 identified)	Single-institution cohort with uniform data collection and pathology confirmation; includes molecular and immunotherapy biomarkers	Small sample size; retrospective design; limited power for statistical inference
OSU CCS Cohort	5 evaluable patients	Captures rare entity with detailed clinical, treatment, and genomic data; includes progression patterns and TTP estimates	Very small cohort; heterogeneity in treatment regimens; outcomes not generalizable
Massoth et al., Oncologist 2021 [7]	21 histiocytic/dendritic tumors	Molecular profiling across DHCS; identifies actionable genomic alterations distinct from follicular dendritic cell sarcomas	Focus on genomic data without correlating with treatment outcomes or clinical responses
Smrke et al., ESMO Open 2022 [8]	55 patients with CCS across multiple centers	Largest international retrospective series on CCS; multi-center design improves generalizability and identifies treatment patterns	Retrospective design; limited molecular data; variability in treatment approaches
Diamond et al., Nature 2019 [17]	18 patients with histiocytic neoplasms	Demonstrates clinical efficacy of MEK inhibition in histiocytic neoplasms with MAPK pathway mutations	Small series with no control group; case-based interpretation; lacks generalizability
Kommalapati et al., Blood 2018 [11]	154 patients with histiocytic sarcoma (SEER-based)	Population-based data allows for estimates of incidence, demographic disparities, and survival outcomes	Registry-based study; lacks treatment details and molecular correlates
Martin-Broto et al., Ann Oncol 2024 [25]	17 patients with CCS in the IMMUNOSARC II trial	Prospective trial data on immunotherapy and targeted agents in CCS; integrates sunitinib and nivolumab activity	Small trial cohort; early-phase design; lacks long-term follow-up data

Abbreviations: DHCS, dendritic and histiocytic cell sarcoma; CCS, clear cell sarcoma; OSU, The Ohio State University; TTP, time to progression; MEK, mitogen-activated protein kinase kinase; MAPK, mitogen-activated protein kinase; SEER, Surveillance, Epidemiology, and End Results; ICI, immune checkpoint inhibitor; PFS, progression-free survival; OS, overall survival

## Data Availability

Data supporting the findings of this study were derived from the electronic health records and institutional pathology and molecular diagnostics systems of The Ohio State University Comprehensive Cancer Center and are not publicly available due to patient privacy and institutional data-use restrictions. De-identified data may be made available from the corresponding author upon reasonable request and with prior approval from the Institutional Review Board and data governance bodies. No new publicly archived datasets were generated in this study.
