# Correction: Tinoco et al. Clear Cell and Histiocytic/Dendritic Cell Sarcomas: Clinical Outcomes, Molecular Features, and Diagnostic Pitfalls. *Cancers* 2026, *18*, 641

**DOI:** 10.3390/cancers18142245

**Published:** 2026-07-14

**Authors:** Gabriel Tinoco, Marium Husain, David Liebner, James L. Chen, Swati Satturwar, Hans Iwenofu, Valerie Grignol, Joal Beane, Scott Lenobel, David Konieczkowski, Carl Quinion, Joel Mayerson

**Affiliations:** 1 Division of Medical Oncology, Department of Internal Medicine, The Ohio State University Comprehensive Cancer Center, Columbus, OH 43210, USA; marium.husain@osumc.edu (M.H.); david.liebner@osumc.edu (D.L.); james.chen@osumc.edu (J.L.C.); 2 Department of Pathology, The Ohio State University Comprehensive Cancer Center, Columbus, OH 43210, USA; swati.satturwar@osumc.edu (S.S.); hans.iwenofu@osumc.edu (H.I.); 3 Division of Surgical Oncology, Department of Surgery, The Ohio State University Comprehensive Cancer Center, Columbus, OH 43210, USA; valerie.grignol@osumc.edu (V.G.);; 4 Department of Radiology, The Ohio State University Comprehensive Cancer Center, Columbus, OH 43210, USA; 5 Department of Radiation Oncology, The Ohio State University Comprehensive Cancer Center, Columbus, OH 43210, USA; 6 Department of Orthopedic Surgery, The Ohio State University Comprehensive Cancer Center, Columbus, OH 43210, USAjoel.mayerson@osumc.edu (J.M.)

## 1. Text Correction

In the original publication [1], there was an error in the Methods, Study Design and Oversight Section, paragraph 1. The following sentences have been deleted because this information is already included in the Institutional Review Board Statement: “Because this was a retrospective review of existing records, the IRB granted a waiver of informed consent and a waiver of HIPAA authorization. All procedures complied with the Declaration of Helsinki and institutional policies on data privacy and security.” 

## 2. Error in Institutional Review Board Statement

In the original publication, there was an error in the Institutional Review Board Statement. The following sentence has been deleted: “Because this was a retrospective review of existing records, the IRB granted a waiver of informed consent and a waiver of HIPAA authorization.” 

## 3. Missing Informed Consent Statement

In the original publication, the Informed Consent Statement was not included. The correct statement is as follows: “Informed Consent Statement: Because this was a retrospective review of existing records, the IRB granted a waiver of informed consent and a waiver of HIPAA authorization.”

The authors state that the scientific conclusions are unaffected. This correction was approved by the Academic Editor. The original publication has also been updated.
